# Evaluation of Pediatric Patients with a Diagnosis of Ureterocele

**DOI:** 10.3390/pediatric14040062

**Published:** 2022-12-07

**Authors:** Özgür Özdemir Şimşek, Sibel Tiryaki, Gökçen Erfidan, Cemaliye Başaran, Seçil Arslansoyu Çamlar, Fatma Mutlubaş, Belde Kasap Demir, Demet Alaygut

**Affiliations:** 1 Department of Pediatric Nephrology, Faculty of Medicine Tepecik Training and Research Hospital, University of Health Sciences, Izmir 35170, Turkey; 2 Department of Pediatric Surgery, Tepecik Training and Research Hospital, University of Health Sciences, Izmir 35170, Turkey; 3 Division of Nephrology, Department of Pediatrics, Tepecik Training and Research Hospital, University of Health Sciences, Izmir 35170, Turkey; 4 Department of Pediatric Nephrology and Rheumatology, Faculty of Medicine, Katip Çelebi University, Izmir 35170, Turkey

**Keywords:** ureterocele, urinary tract infection, vesicoureteral reflux, voiding cystourethrogram

## Abstract

Aim: The presence and clinical importance of vesicoureteric reflux in patients with a double collecting system are being questioned. Therefore, the role of voiding cystourethrography in the management of patients with ureterocele is unclear. This study aimed to evaluate patients with a ureterocele in terms of urinary tract infection (UTI) and vesicoureteral reflux (VUR). Material Methods: The cases who were admitted to the Pediatric Nephrology Clinic of Health Sciences University Tepecik Training and Research Hospital between 2012 and 2022 and were diagnosed with ureterocele were evaluated retrospectively. Demographic, clinical, and laboratory data were obtained from file records. Results: All patients diagnosed with ureterocele and voiding cystourethrography (VCUG) were evaluated. A total of 24 (female 13 (54.2%)) children were included. The reasons for admission were antenatal hydronephrosis in 13 (54.2%) patients, UTI in 9 (37.5%) patients, and incidentally diagnosed ureterocele in 2 (8.3%) patients. Urinary tract infection was observed in 20 patients at admission, recurrent UTI in 21 patients at follow-up, preoperative pyelonephritis in 12 patients. VUR was found in 11 patients, and severe VUR (≥stage 3) was found in 9 patients. Ten patients had ipsilateral hydronephrosis, and 14 patients had a double collecting system. The presence of VUR was found to be associated with female gender, UTI at admission, and recurrent UTI at follow-up (*p* < 0.05). However, there was no difference between groups with or without VUR in terms of ipsilateral hydronephrosis, scar formation, and the need for surgery (*p* > 0.05). Conclusions: We could not demonstrate any criteria to select patients to receive VCUG; on the other hand, VUR did not cause more kidney damage. Our study supports the need for more scientific data to determine management in patients with ureterocele.

## 1. Introduction

A ureterocele is an enlargement of the terminal ureter within the bladder and/or urethra. It is an ectopic form of the distal part of the ureter that extends to the bladder, bladder neck, or urethra [1]. Diagnosis rates of congenital urinary anomalies have increased with the use and accessibility of antenatal ultrasonography (USG). With antenatal USG in patients with ureterocele, most patients with obstruction are diagnosed and confirmed prenatally. In cases without an antenatal diagnosis, ureterocele can be diagnosed in a wide age range, from the first days of life to advanced decades. While patients present with symptoms such as obstruction and urinary tract infection (UTI) in the early period, they may present with recurrent UTI, kidney stones, pain, urinary incontinence, and voiding disorders in the following periods [1]. Ureterocele may be associated with single or double collecting systems. About 80% of ureteroceles are associated with the double collecting system and originate from the upper pole ureter. Notably, 60% of these are ectopic [2]. According to the classification system used by the American Academy of Pediatrics, ureteroceles are divided into two classes: intravesical ureteroceles that are entirely within the bladder, and ectopic ureteroceles, some of which extend beyond the bladder neck into the urethra [3].

After the diagnosis, a treatment plan is made according to the anatomical and functional evaluation of the affected structures. As ureteroceles differ in terms of age and clinic, they also differ in terms of diagnosis and treatment. The main purpose is to protect the kidney function; eliminate existing findings, such as infection, obstruction, reflux, and urinary incontinence; and provide urinary continence [4]. Imaging of patients with ureterocele starts with USG. Radionuclide renal imaging, voiding cystourethrogram (VCUG), intravenous pyelogram (IVP), magnetic resonance urography (MRU), and endoscopy can also be used to reveal anatomy and plan management. The aim of this study is to evaluate the diagnosis and follow-up of patients with ureterocele followed in our clinic focusing on the place of VCUG.

## 2. Material-Methods

The study was planned as a retrospective descriptive study that included pediatric patients aged 0–18 years who were followed up in the Pediatric Nephrology Clinic of the University of Health Sciences and diagnosed with ureterocele between 2012 and 2022. The study was approved by the local ethics committee (Decision No: 2022/06–19, Date: 15 June 2022). The following data were reviewed: gender, age at diagnosis, complaint at presentation, the presence of UTI at diagnosis, the presence of recurrent UTI, pyelonephritis history, and whether VCUG was performed. The presence and grade of vesicoureteral reflux (VUR), the presence of double collecting system, first and last technetium-99m dimercaptosuccinic acid (DMSA), progression, the need for (and reasons for) surgery, how many (and which) surgeries the patient had, and the patient’s post-operative UTI history were evaluated.

The SPSS package program (IBM SPSS Statistics for Windows, version 25.0. Armonk, NY: IBM Corp, 2017) was used for statistical analyses. Variables with normal distribution are shown as mean values ± standard deviation (SD), variables with abnormal distribution are shown as median (range), and the rest are expressed as frequency. The chi-square test was used to compare categorical variables between groups. The Kolmogorov–Smirnov test was used to evaluate the normal distribution of continuous variables between groups. All parameters were distributed abnormally, so they were evaluated by the Mann–Whitney U test. For this study, *p* < 0.05 was considered statistically significant.

## 3. Results

There were 29 patients who were followed up for ureterocele in the specified period. Among these, 24 patients had full medical records and underwent VCUG. The demographic and clinical data of the whole group are depicted in Table 1. When the presenting symptoms of the patients were evaluated, antenatal hydronephrosis was found in 13 (54%) patients, UTI in 9 (38%) patients, and ureterocele incidentally in 2 (8%) patients. UTI was present in 20 (83.3%) patients at the time of diagnosis. Twenty-one (87.5%) patients had recurrent UTIs at follow-up. Twelve (50%) of twenty-four patients had a history of preoperative pyelonephritis.

When sonographies were evaluated, 14 had a double collecting system. Among the 24 patients with VCUG, 11 had VUR (all with a double collecting system). Six of these had ipsilateral hydronephrosis. On the other hand, four patients with no VUR had hydronephrosis (*p* = 0.615). Nine patients had severe VUR (≥stage 3), and five of these had ipsilateral hydronephrosis (*p* = 0.285).

Patients with a double collecting system were then evaluated in terms of the detection and outcome of reflux (Table 2). VUR was more frequent in girls but not associated with any complaints (*p* = 0.078, including UTI *p* = 0.287), sonography, or scintigraphy findings at the presentation (Table 2). When follow-up was evaluated, recurrent UTI, pyelonephritis, and need for surgery were more frequent in patients with VUR, but there was no difference in scar progression rate (Table 2).

Only one patient did not require surgery in the follow-up. Indications for surgery were UTI in 22 patients and hydronephrosis secondary to obstruction in 1 patient. Ureterocele incision was performed in 12 patients, ureterocele incision and partial nephrectomy in 7 patients, and ureterocele incision and ureteroneocystostomy in 4 patients. Among these, 11 underwent multiple surgeries. There was also no difference between groups with or without VUR in terms of the need for multiple surgeries (*p* = 0.837). Postoperative UTIs were seen in only 3 (13%) of 23 operated patients. All patients had unilateral disease, and none had chronic kidney disease in the follow-up. Creatinine, eGFR, weight, and height at the last visit are shown in Table 1.

## 4. Discussion

Each ureterocele patient is unique with a wide spectrum of anatomical variations. The presence of VUR or obstruction mainly alter the prognosis, but even presentation is variable. Admission with antenatal hydronephrosis constitutes the majority; however, UTI or obstruction is not rare. UTI can occur at any age with a highly variable clinical presentation, and early diagnosis and treatment are vital to prevent future complications and morbidities [5]. Therefore, imaging provides valuable data in patients with ureterocele. However, designating an algorithm is hard due to the highly variable nature of the abnormality.

Most of the urinary congenital abnormalities are diagnosed with antenatal USG, one of which is duplex system anomalies. Many studies in the literature show that approximately 70% of patients diagnosed with the prenatal duplex system are associated with ureterocele [6]. The presence of a duplex system presents questions surrounding VUR. DMSA is the gold standard for the evaluation of renal function in patients with ureterocele and is one of the most important imaging modalities in follow-up [7]. VCUG is mostly recommended to evaluate VUR, but its necessity is questioned in regard to radiation exposure and the need for bladder catheterization [8]. Contrast-enhanced voiding urosonography (ceVUS) is a method based on evaluating contrast reflux at the time of voiding. There is no radiation exposure, since it is an ultrasonographic evaluation. However, it requires experience in the subject and specific contrast material; therefore, it cannot be performed in every center [9]. It can also not be performed in our center.

A study evaluating USG, VCUG, and DMSA findings after UTI retrospectively found that normal USG findings can exclude the diagnosis of severe VUR in patients with UTI and mild renal scarring. The authors recommended avoiding VCUG if there is no additional predisposing factor [10]. On the other hand, another study evaluating 93 patients with antenatal suspicion of renal anomaly found that the diagnosis of VUR could not be excluded from normal USG findings [11]. We reviewed our patient records to reveal any criteria to select patients to receive VCUG. Our data showed no difference comparing patients with or without VUR in terms of clinical or radiological findings at presentation. We also looked for a relationship between high-grade reflux and hydronephrosis but found none.

Then we evaluated if the presence of VUR altered the prognosis. Patients with VUR experienced more UTIs, including pyelonephritis, and underwent more surgeries, but looking at the final outcome, there was no difference in terms of scar progression.

The main limitations of our study are the retrospectively collected data and the limited number of patients. On the other hand, our study contributes to current literature questioning the place of VCUG in patients with ureterocele. Our findings support the lack of evidence for the need for VCUG in every patient with ureterocele but failed to reveal any selection criteria.

## 5. Conclusions

Ureterocele can present with various clinical findings at any time of life. It is a congenital abnormality that can sometimes show symptoms for the first time in the adult age group and sometimes cannot be diagnosed at all. Diagnosis is usually made by USG, and symptoms are not always correlated with radiological findings, so the place of further investigations such as VCUG are questioned. Our study supports the need for more scientific data to determine management in patients with ureterocele.

## Figures and Tables

**Table 1:** Demographic and clinical data of patients with ureterocele.

Patients	24 Cases with Ureterocele
Gender	
Female	13 (54.2%)
Male	11 (45.8%)
Age of admission (months) (median)	1.5 (0–66)
Follow-up time (months) (median)	37.5 (1–160)
Height (cm) (mean ± SD *)	104 ± 31
Height percentile (median)	62 (15–98)
Weight (kg) (median)	15.5 (6–72)
Weight percentile (median)	70 (23–98)
Serum creatinine (mg/dL) (median)	0.40 (0.3–0.7)
eGFR ** mL/min per 1.73 m^2^ (mean ± SD)	111 ± 22

* SD: standard deviation; ** eGFR values were calculated using height and creatinine values according to Schwarz.

**Table 2:** Comparing patients with or without VUR among 14 with a double collecting system.

	**Patients (n = 14)**	**At Presentation**					
		**Age of Diagnosis (Months)**	**Follow-Up Time**	**Gender (Female%)**	**UTI**	**HN**	**Scar in the First DMSA**
With VUR	11	10 (0–66)	41 (3–60)	8 (72%)	10 (90%)	6 (54%)	8 (72%)
Without VUR	3	0	39 (2–80)	0	2 (66.6%)	2 (66.6%)	2 (66.6%)
*p*		0.659 ^a^	0.126 ^a^	0.024 ^b^	0.287 ^b^	0.615 ^b^	0.125 ^b^
	**Patients (n = 14)**	**In Follow-Up**					
		**Recurrent UTI**	**Pyelonephritis**	**New Scars**	**Need for Surgery**		
With VUR	11	11 (100%)	8 (72%)	4 (36%)	11 (100%)		
Without VUR	3	2 (66.6%)	0	2 (66.6%)	2 (66.6%)		
*p*		0.047 ^b^	0.024 ^b^	0.408 ^b^	0.047 ^b^		

Statistical method: ^a^ Mann–Whitney U test, ^b^ Chi-square; DMSA: technetium-99m dimercaptosuccinic acid; HN: hydronephrosis; UTI: urinary tract infection; VUR: vesicoureteral reflux.

## Data Availability

Data available on request due to restrictions eg privacy or ethical. The data presented in this study are available on request from the corresponding author. Data are not publicly available due to hospital rules.
